# Performance Evaluation of Volumetric Water Content and Relative Permittivity Models

**DOI:** 10.1155/2013/421762

**Published:** 2013-10-24

**Authors:** Muhammad Mukhlisin, Almushfi Saputra

**Affiliations:** ^1^Department of Civil and Structural Engineering, Faculty of Engineering and Built Environment, Universiti Kebangsaan Malaysia, 43600 Bangi, Selangor, Malaysia; ^2^Department of Civil Engineering, Polytechnic Negeri Semarang, Jl. Prof. Soedarto, SH, Tembalang, Semarang 50275, Indonesia

## Abstract

In recent years many models have been proposed for measuring soil water content (*θ*) based on the permittivity (*ε*) value. Permittivity is one of the properties used to determine *θ* in measurements using the electromagnetic method. This method is widely used due to quite substantial differences in values of *ε* for air, soil, and water, as it allows the *θ* value to be measured accurately. The performance of six proposed models with one parameter (i.e., permittivity) and five proposed models with two or more parameters (i.e., permittivity, porosity, and dry bulk density of soil) is discussed and evaluated. Secondary data obtained from previous studies are used for comparison to calibrate and evaluate the models. The results show that the models with one parameter proposed by Roth et al. (1992) and Topp et al. (1980) have the greatest *R* ^2^ data errors, while for the model with two parameters, the model proposed by Malicki et al. (1996) agrees very well with the data compared with other models.

## 1. Introduction

Measurement of water content (*θ*) in soil has become a major component of the various fields of geotechnical analysis. Measurement of *θ* is needed to support many bodies of research related to the soil [1]. For example, in agriculture, *θ* is an important factor for irrigation and crop quality maintenance. On the other hand, in hydrology, determining the rate and quantity of water movement in soil requires the *θ* measurement. Meanwhile, in forestry, *θ* is required for information on the water storage capacity of soil. Besides, *θ* also affects the stability of the slope of soil due to its relationship with the soil strength [2–9].

Measurements of *θ* can be categorised as direct and indirect measurements. Gravimetric measurement is a direct measurement which is categorised as a conventional method. In this method, the value of *θ* is determined by subtracting dry from wet soil sample weights. This method is very accurate but it is not practical due to the long time it takes to get the result. However this method is used as a calibration for other techniques.

For indirect measurement, electrical methods for measuring *θ* have primarily been subjected to extensive study due to their ease and practicality of use. These methods have been widely used and discussed in many previous studies (e.g., [10–14]). Moreover, issues of the instruments used for measurement of water content, from small-scale (<1 m^2^) to large-scale (100 m^2^), and suitable methods for measurement at those various scales have also been discussed in detail by [15]. In their study, Robinson et al. [15] concluded that the method for measuring water content requires improvement. In some other studies (e.g., [16–18]) electrical properties are measured to get the characteristic of *θ*. In their study it can be seen that permittivity (*ε*) measurement can be used to predict *θ*. Permittivity (*ε*) is the most common of electrical properties that are used to measure *θ*. Although there are also some techniques by measuring the capacitance of soil, then it converted into *ε* (e.g., [19, 20]).

To represent the *ε*-*θ* relationship, there are several models that have been proposed in the last few decades (e.g., [14, 18–30]). To produce the *ε*-*θ* relationship model, most of them used gravimetric measurement for data calibrations. However, data from previous studies were also used (e.g., [25]).

All models can be categorised as having one parameter or two or more parameters. Models with one parameter only involve the relationship between the permittivity and water content, whereas models with two or more parameters include other parameters such as porosity or dry bulk density. This classification is used to analyse the influence of parameters other than the permittivity that affect the value of water content, and no previous study has tried to analyse this. 

In this study, the models proposed by [14, 18–23, 25, 28–30] were reviewed and compared using secondary data from previous studies (e.g., [22, 27, 31, 32]). The data are then used to determine which model has a significant *ε*-*θ* relationship.

## 2. Theory and Method

Many equations have been proposed for calibration of *ε* and *θ*. These models can be divided into two categories: models with one parameter and models with two or more parameters.

### 2.1. Model with One Parameter

There are some proposed models that show the *ε*-*θ* relationship. Topp et al. [22] successfully introduced the *ε*-*θ* relationship that is commonly used in the geotechnical area for the first time. The relationship is

(1a) $$\begin{array}{c} \varepsilon =3.03+9.3\theta +146.0\theta^{2}-76.7\theta^{3}, \end{array}$$

where *ε* is the relative permittivity or dielectric constant and *θ* is the volumetric water content of the soil. The experiment uses Time Domain Reflectometry (TDR) at a frequency between 1 MHz and 1 GHz to measure *ε* of several mineral soils. Then a polynomial fitting is used empirically to obtain the *ε*-*θ* relationship model. The estimated error in this model is 0.013 [22]. In their study, Topp et al. [22] also provide another form of (1a) as follows:

(1b) θ=−5.3×10−2+2.92×10−2ε−5.5 ×10−4ε2+4.3×10−6ε3.

The *ε*-*θ* relationship models for organic soil and 450 *μ*m glass beads are also shown as follows:

(1c) $$\begin{array}{c} \varepsilon =1.74-0.34\theta +135\theta^{2}-55.3\theta^{3},\;\text{organic}\;\;\text{soil} \end{array}$$

(1d) $$\begin{array}{c} \varepsilon =3.57+31.7\theta +114\theta^{2}-68.2\theta^{3},\;450\;\mu \text{m}\;\;\text{glass}\;\;\text{beads}. \end{array}$$

Roth et al. [21] used miniprobe TDR for their experiment to propose another empirical equation for the *ε*-*θ* relationship, which had been used previously by [33]. The *ε*-*θ* relationship for mineral soil proposed by [21] is

(2a) $$\begin{array}{c} \theta =-0.0728+0.0448\varepsilon -0.00195\varepsilon^{2}+0.0000361\varepsilon^{3}, \end{array}$$

while the *ε*-*θ* relationship for organic soil and material is

(2b) $$\begin{array}{c} \theta =-0.0233+0.0285\varepsilon -0.000431\varepsilon^{2}+0.00000304\varepsilon^{3}. \end{array}$$

The error estimations of these equations for mineral soil and organic soil are 0.015 and 0.035 cm^3^ cm^−3^, respectively [21].

Ferré et al. [25] proposed a simple equation for the *ε*-*θ* relationship. This equation was generated from the principle of dielectric mixing models and using TDR without coatings:

(3) $$\begin{array}{c} \theta =0.1181\sqrt{\varepsilon }-0.1841. \end{array}$$

A simple equation was also introduced by [29]. They used 505 measurements from organic forest floor sample experiments using TDR, where the *ε*-*θ* relationship is

(4) $$\begin{array}{c} \theta =0.136\sqrt{\varepsilon }-0.119. \end{array}$$

A coaxial transmission system at a frequency of 100 MHz was used by [18] to produce another model of the *ε*-*θ* relationship, which used a wide range of soil textures samples:

(5) $$\begin{array}{c} \theta =-0.0286+0.02435\varepsilon -0.0003421\varepsilon^{2}+0.00000237\varepsilon^{3}. \end{array}$$

Permittivity based on capacitance measurement was investigated by [20]. They proposed an empirical model from experiment using a type of quartz sand with particle sizes in the range 0.15–0.9 mm:

(6) $$\begin{array}{c} \varepsilon =A{\left(\frac{1}{1+{\left(\alpha \left(1-\theta \right)\right)}^{n}}\right)}^{1-(1/n)}+B, \end{array}$$

where *A* = 33, *B* = 2, *α* = 1.5, and *n* = 14.

### 2.2. Model with Two or More Parameters

Some relationship equations for permittivity and soil water content were also influenced by other parameters such as porosity and bulk density. By using the concept of mixing models and using data from other studies (e.g., [34–36]), Wang and Schmugge [30] proposed the following equations:

(7a) $$\begin{array}{c} \varepsilon =\theta \left(\varepsilon_{i}+\left(\varepsilon_{w}-\varepsilon_{i}\right)\frac{\theta }{\theta_{t}}\gamma \right)+\left(\eta -\theta \right)\varepsilon_{a}+\left(1-\eta \right)\varepsilon_{r}. \end{array}$$

Equation (7a) is used for *θ* ≤ *θ*_*t*_, while for *θ* > *θ*_*t*_ the following equation is used:

(7b) $$\begin{array}{c} \varepsilon =\theta_{t}\left(\varepsilon_{i}+\left(\varepsilon_{w}-\varepsilon_{i}\right)\gamma \right)+\left(\theta -\theta_{t}\right)\varepsilon_{w}+\left(\eta -\theta \right)\varepsilon_{a}+\left(1-\eta \right)\varepsilon_{r}, \end{array}$$

where *ε*_*i*_, *ε*_*w*_, *ε*_*a*_, and *ε*_*r*_ are the permittivity of ice, water, air, and rock, respectively (i.e., *ε*_*i*_ = 3.2, *ε*_*w*_ = 80, and *ε*_*a*_ = 1), while *θ*_*t*_ is the transition moisture (0.16–0.33), *η* is the porosity of soil (0.5), and *γ* is the fitting parameter (0.3–0.5) [30].

Roth et al. [28] proposed the equation based on the dielectric mixing model which has been described by [24]. The experiments were carried out by measuring a wide range of soil types using TDR with the error value of soil water content, no more than 0.013 cm^3^ cm^−3^ [28], with forms of the following equation:

(8a) $$\begin{array}{c} \theta =\frac{\varepsilon^{\gamma }-\left(1-\eta \right)\varepsilon_{s}^{\gamma }-\eta \varepsilon_{a}^{\gamma }}{\varepsilon_{w}^{\gamma }-\varepsilon_{a}^{\gamma }};\;\gamma =-1, \end{array}$$

(8b) $$\begin{array}{c} \theta =\frac{\varepsilon^{\gamma }-\left(1-\eta \right)\varepsilon_{s}^{\gamma }-\eta \varepsilon_{a}^{\gamma }}{\varepsilon_{w}^{\gamma }-\varepsilon_{a}^{\gamma }};\;\gamma =1, \end{array}$$

where *γ* = −1 for three phases in series and *γ* = 1 for three phases in parallel.

Another model was proposed by [23]. They conducted experiments using TDR and 62 kinds of soil samples consisting of mineral soils, organic soil, standard pot soils, artificial peat-loess and peat-sand, sea and river sand, forest litter, and so forth, which differ in terms of texture and bulk density, which gives an uncertainty of soil water content of 0.03 [23]:

(9) $$\begin{array}{c} \theta =\frac{\sqrt{\varepsilon }-3.47+6.22\eta -3.82\eta^{2}}{7.01+6.89\eta -7.83\eta^{2}}. \end{array}$$

Gardner et al. [19] used capacitance measurement methods to obtain soil water content with soil dry bulk density values ranging rom 1.08 to 1.49 and then used multiple linear regression analysis to best fit the measurement data, resulting in the following equation:

(10) $$\begin{array}{c} \theta =\frac{\sqrt{\varepsilon }+1.208-2.454\rho }{9.93}, \end{array}$$

where *ρ* is dry bulk density.

Robinson et al. [14] developed an equation for coarse textured, layered soils by using TDR and coarse-grained, glass bead, and quartz grains:

(11) $$\begin{array}{c} \theta =\eta \left(\frac{\sqrt{\varepsilon }-\sqrt{\varepsilon_{\text{dry}}}}{\sqrt{\varepsilon_{\mathrm{sat}}}-\sqrt{\varepsilon_{\text{dry}}}}\right), \end{array}$$

where *ε*_dry_ and *ε*_sat⁡_ are the permittivity values for dry and saturated soil, respectively.

Table 1 shows the 11 proposed equations of the *ε*-*θ* relationship for one and two or more parameters. It provides a brief explanation, including the experimental method, soil type, properties of the soil, and the sources information for each proposed equation.

Models with one parameter use only a single parameter or variable to calculate soil moisture content. This parameter is permittivity. Most of these models are defined using empirical methods. Models with two or more parameters have other parameters besides permittivity, such as porosity and bulk density. 

### 2.3. Secondary Data

In this study, reference data are needed to test the ability of all these models. For this purpose, secondary data obtained from previous experimental studies that showed the relationship between soil water content and permittivity were used. Overall, there are 44 secondary data, and the data sources and soil porosity used in this study can be seen in Table 2. These data were generated from experiments using different methods such as TDR (e.g., [14, 21, 22, 26, 28, 31, 32]), capacitance probe [20], and frequency domain [27] and also from varying types of soil. 

The value of relative permittivity air, water, dry soil, and saturated soil can be seen in Table 3. Relative permittivity of dry soil and saturated soil is obtained from the study of the previous researches [14]. Relative permittivity of material is affected by the chemical components of its constituent and can be calculated by using the mixture model [37]. 

## 3. Results and Discussion

**Figure 1:**
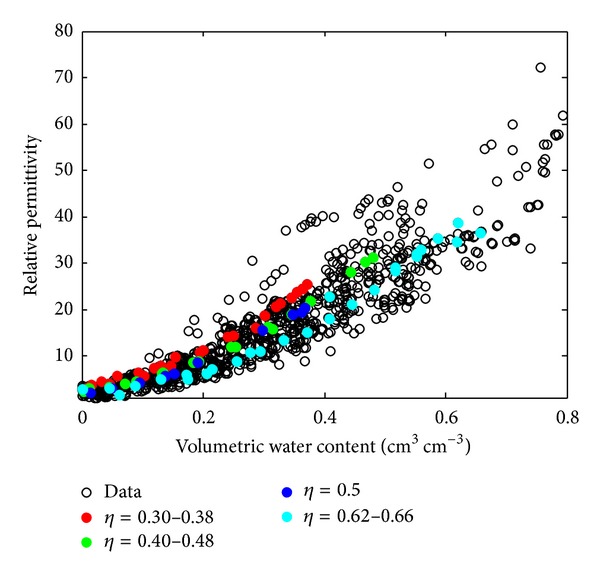
Secondary data of volumetric water content and permittivity as a function of porosity.

The effects of porosity on the *ε*-*θ* relationship are shown in Figure 1. Four secondary data samples which have porosity ranging from 0.30 to 0.66 are highlighted.

Figure 1 shows that the smaller the soil porosity, the greater the value of permittivity for a given value of volumetric water content. In this condition, the pores in the soil will be filled by water and air. Therefore when the porosity is large, then the rest of the pores are filled by air. This corresponds to the concept of dielectric mixing used in models by [24]. When most of the volume fraction of soil pores is filled by air, it donates a small value of the total permittivity of the soil. This figure also shows that the spread of data does not occur significantly for small water content (0–0.1). In this condition, the value of permittivity is in the range of 1–5. Otherwise, when the water content begins to increase, it produces scattered data values.

### 3.1. Model with One Parameter

**Figure 2:**
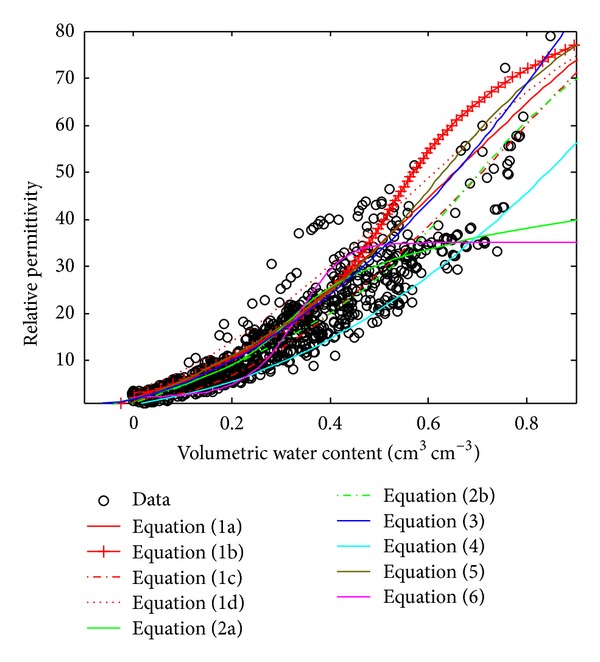
Comparisons using all data for (1a) to (6).

Figure 2 shows curves for (1a) to (6), which have one parameter. All equations appeared to cover all of the available data. However, each equation appears in a certain position within the data. Equations (1a), (1b), (1d), (3), and (5) are quite close to each other and tend towards the upper part of the data which have relatively small porosity (<0.5). On the other hand, the larger porosity (>0.6) is occupied by (4). 

Almost all of the curves show a similar trend, except for (2a) and (6). The curve for (2a) indicates a lower increase in permittivity when the water content is greater than 0.4, while the curve for (6) gives a constant permittivity value when the water content is greater than 0.4. Wu et al. [20] explain that this reduction in the increase of permittivity is due to the effect of saturation in the soil. 

Equations (1c) and (2b) occupy the central part of data distribution. These curves provide a reasonably safe prediction of the *ε*-*θ* relationship for equations with one parameter or without any parameters of porosity.  Table 4 shows the *R*-square of each curve to data and also the Root Mean Square Error (RMSE) of each equation to data. Equation (2b) gives a better result for *R*-square and RMSE compared with other equations with one parameter.

### 3.2. Model with Two Parameters

**Figure 3:**
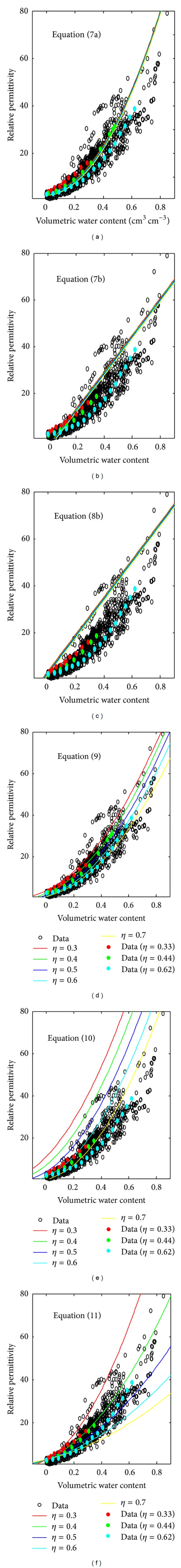
Comparison of (7a) to (11) with all data and different porosity.

Figure 3 shows the effect of porosity (*η* = 0.3 to *η* = 0.7) on the suitability of (7a) to (11) with data and also displays some of the data with a value of porosity (0.33, 0.44, and 0.62) in order to see the fit between data with a curve based on the value of porosity. In Figure 3(a), it can be seen that (7a) only fits in a certain small area of the data, though with different porosity. In this equation, the effect of changing porosity is not significant. Figures 3(b) and 3(c), with all the possible values of porosity, show that neither of these equations is quite enough to follow the pattern of the data. In these equations, the trend is linear for both of the graphs.

A better plot is shown in Figure 3(d), where the equation occupies all of the data well. This figure shows that (9), which was proposed by [23], has a significant effect on changing porosity. The figure also shows that curves merge very well in the range of the secondary data. 

Overestimated results are produced in Figure 3(e). Curves with small porosity parameters can not even cover the area of data. There are only two curves passing through the area of data. Nevertheless, these curves are inconsistent with the position of the porosity of the data.

Figure 3(f) shows a wide spread of curves for changes of porosity as the water content increases. Almost all areas are covered except data for water content values smaller than 0.2. However, when viewed in terms of the porosity data, the curves in this figure do not look quite as good because they spread without following the porosity data. 

## 4. Conclusion

A comparison of some equations for the *ε*-*θ* relationship was performed to provide an overview of the efficacy and ability of each equation in describing the *ε*-*θ* relationship and its correlation to the porosity of soil. In this study, secondary data were used as a reference to compare the equations. Secondary data with porosity values were plotted in one graph to show the effect of soil porosity on the relationship between water content and permittivity. For the same water content, the permittivity of soil decreases with increasing porosity. In this case, porosity should be taken into account when considering the *ε*-*θ* relationship.

There are some models of the *ε*-*θ* relationship that do not fit with data in a certain range. For equations with one parameter, the model of [21] for mineral soil and the model of [20] indicate curves which tend to slow down the increase in permittivity at near saturation conditions of soil. Furthermore, equations with one parameter are also not able to cover all areas of the data.

However, of the models that included a porosity parameter, apparently not all of them could explain the effect of porosity on the *ε*-*θ* relationship very well. From this study, only the equation proposed by [23] gives a fairly good conformity of data for different porosity values of soil.

## Figures and Tables

**Table 1:** Summary of all equations of *ε*-*θ* relationship.

Equations	Source	Experimental method	Soil type	Properties of soil		
				Porosity (cm^3^cm^−3^)	Bulk density (g cm^−3^)	Particle density (g cm^−3^)
Model with one parameter						
(1a)			(i) Mineral soil (ii) Organic soil (iii) Vermiculite (iv) Glass beads	—	(i) 1.04–1.44 (ii) 0.422 (iii) 1.08 (iv) 1.49–1.61	—
(1b)	Topp et al. [22]	*ε*: using TDR Tektronik Model 7S12 to perform 18 experiments with different treatments *θ*: using gravimetric technique				
(1c)			Organic soil	—	0.422	—
(1d)			450 *μ*m glass beads	—	1.60–1.61	—
(2a)	Roth et al. [21]	*ε*: TDR miniprobe 250 ps rise time needle pulse *θ*: gravimetric technique	9 Mineral soils	0.418–0.482	1.26–1.55	2.28–2.67
(2b)			7 Organic soils	0.527–0.785	0.2–0.77	0.70–1.63
(3)	Ferré et al. [25]	Using model of inverse averaging for TDR method by analysing the mixing model	—	—	—	—
(4)	Schaap et al. [29]	*ε*: TDR Tektronix 1502B *θ*: gravimetric technique	25 samples of forest floors	—	0.086–0.263	1.3
(5)	Curtis [18]	Coaxial Transmission/reflection apparatus controlled by a Hewlett-Packard 8510C Vector Network Analyzer system 45 MHz to 26.5 GHz	—	—	—	—
(6)	Wu et al. [20]	*ε*: based on capacitance measurement *θ*: gravimetric technique	Quartz sand	—	—	—

Model with two parameters						
(7a)	Wang and Schmugge [30]	Modelling using data from other studies [34–36]	22 different samples	0.4–0.6	1.1–1.7	2.6–2.75
(7b)						
(8a)	Roth et al. [21]	TDR	From 11 different field sites			
(8b)						
(9)	Malicki et al. [23]	TDR CAMI	62 kinds of soil samples	0.33–0.95	0.13–1.66	1.06–2.7
(10)	Gardner et al. [19]	Capacitance probe 80–150 MHz	(i) Brown earths (ii) Silica materials	—	(i) 1.08–1.49 (ii) 1.24–1.63	—
(11)	Robinson et al. [14]	TDR Tektronix 1502B	Coarse grained, quartz grain, sandy soil	—	—	—

**Table 2:** Source of secondary data and porosity.

Porosity	Data source
0.30–0.38	Friedman [26]; Sabouroux and Ba [31]
0.40–0.48	Friedman [26]; Hilhorst et al. [27]
0.5	Skierucha et al. [32]
0.62–0.66	Roth et al. [21]; Friedman [26]
—	Topp et al. [22]; Malicki et al. [23]; Robinson et al. [14]; Curtis [18]; Gardner et al. [19]; Dobson et al. [24]; Wang and Schmugge [30]

**Table 3:** Relative permittivity of material properties.

Material	Relative permittivity	Chemical elements
Air (*ε*_air_)	1	N_2_, O_2_
Water (*ε*_water_)	80	H_2_O
Dry soil (*ε*_dry soil_)	2–4	N, P, K, Ca, Mg, S, Cu, Zn, Fe, Mn, B, Cl, Na, H
Saturated soil (*ε*_saturated soil_)	23–28	H_2_O, N, P, K, Ca, Mg, S, Cu, Zn, Fe, Mn, B, Cl, Na, H

**Table 4:** *R*-square and root mean square error (RMSE) of the equations to data.

Equations	(1a)	(1b)	(1c)	(1d)	(2a)	(2b)	(3)	(4)	(5)	(6)
*R* ^2^	0.749	0.8189	0.824	0.480	0.807	0.877	0.834	0.612	0.834	0.705
RMSE	6.131	0.078	5.133	8.824	0.071	0.058	0.075	0.114	0.075	6.536
